# Evaluating machine learning enhanced intelligent‐optimization‐engine (IOE) performance for ethos head‐and‐neck (HN) plan generation

**DOI:** 10.1002/acm2.13950

**Published:** 2023-03-06

**Authors:** Justin Visak, Enobong Inam, Boyu Meng, Siqiu Wang, David Parsons, Dan Nyugen, Tingliang Zhang, Dominic Moon, Vladimir Avkshtol, Steve Jiang, David Sher, Mu‐Han Lin

**Affiliations:** ^1^ Department of Radiation Oncology University of Texas Southwestern Medical Center Dallas Texas USA; ^2^ Medical Artificial Intelligence and Automation Laboratory, Department of Radiation Oncology University of Texas Southwestern Medical Center Dallas Texas USA

**Keywords:** adaptive radiotherapy, head & neck, machine‐learning

## Abstract

**Purpose:**

Varian Ethos utilizes novel intelligent‐optimization‐engine (IOE) designed to automate the planning. However, this introduced a black box approach to plan optimization and challenge for planners to improve plan quality. This study aims to evaluate machine‐learning‐guided initial reference plan generation approaches for head & neck (H&N) adaptive radiotherapy (ART).

**Methods:**

Twenty previously treated patients treated on C‐arm/Ring‐mounted were retroactively re‐planned in the Ethos planning system using a fixed 18‐beam intensity‐modulated radiotherapy (IMRT) template. Clinical goals for IOE input were generated using (1) in‐house deep‐learning 3D‐dose predictor (AI‐Guided) (2) commercial knowledge‐based planning (KBP) model with universal RTOG‐based population criteria (KBP‐RTOG) and (3) an RTOG‐based constraint template only (RTOG) for in‐depth analysis of IOE sensitivity. Similar training data was utilized for both models. Plans were optimized until their respective criteria were achieved or DVH‐estimation band was satisfied. Plans were normalized such that the highest PTV dose level received 95% coverage. Target coverage, high‐impact organs‐at‐risk (OAR) and plan deliverability was assessed in comparison to clinical (benchmark) plans. Statistical significance was evaluated using a paired two‐tailed student *t*‐test.

**Results:**

AI‐guided plans were superior to both KBP‐RTOG and RTOG‐only plans with respect to clinical benchmark cases. Overall, OAR doses were comparable or improved with AI‐guided plans versus benchmark, while they increased with KBP‐RTOG and RTOG plans. However, all plans generally satisfied the RTOG criteria. Heterogeneity Index (HI) was on average <1.07 for all plans. Average modulation factor was 12.2 ± 1.9 (*p* = n.s), 13.1 ± 1.4 (*p* = <0.001), 11.5 ± 1.3 (*p* = n.s.) and 12.2 ± 1.9 for KBP‐RTOG, AI‐Guided, RTOG and benchmark plans, respectively.

**Conclusion:**

AI‐guided plans were the highest quality. Both KBP‐enabled and RTOG‐only plans are feasible approaches as clinics adopt ART workflows. Similar to constrained optimization, the IOE is sensitive to clinical input goals and we recommend comparable input to an institution's planning directive dosimetric criteria.

## 1 INTRODUCTION

External beam radiotherapy (RT) is a staple of head and neck (H&N) cancer management for both definitive and post‐operative settings. Historically, these treatments were planned used 3‐dimensional conformal radiotherapy (3DCRT) that limited dose escalation and organs‐at‐risk (OAR) sparing.^1^, ^2^ More recently, H&N treatments are planned using intensity‐modulated radiotherapy (IMRT)^1^, ^3^ or volumetric modulated arc therapy (VMAT)^4^, ^5^ which allows for multi‐level simultaneous integrated boost treatment schemata and enhances OAR sparing. Generally, H&N RT planning is regarded as the most complex because of the proximity and quantity of higher‐impact dose‐limiting organs (DLO) to the multi‐dose level targets. IMRT and VMAT methods attempt to ease this difficulty by utilizing an inverse planning optimization process that allows the planner direct input to the hyper‐parameters and their relative weights.^6^ While this enables the planner to produce higher‐quality plans than 3DCRT, inverse planning is an iterative "trial‐and‐error" approach and heavily depends on a planner's skill and experience.^7^ Moreover, it is nearly impossible for the planner to predict the physician's optimal target‐OAR tradeoffs that may lead to several rounds of review despite the planner satisfying the original dosimetric criteria. These dosimetric criteria are typically generated based on published, population‐based metrics, such as Radiation Therapy Oncology Group (RTOG) reports and physician experience.

Throughout the several weeks of daily treatment, a H&N patient may experience anatomical changes that causes the meticulously created reference plan to become suboptimal for further treatment. An emerging clinical practice, adaptive radiotherapy (ART), attempts to address these potential issues on either an offline or online time scale.^8^ In order to have a successful online adaptation session, a robust reference plan must first be generated. Online adaptive therapy poses the most technical challenges on an institution and staff as it requires every step of the conventional treatment planning process to be compressed into under an hour. This time‐scale compression is necessary in order to facilitate a comfortable treatment for the patient and minimize intrafractional motion. During online ART, the patient remains on the table as daily images are acquired and a new plan is generated including all physics quality assurance checks. Performing this workflow was nearly infeasible until recent commercial online adaptive therapy systems came to market. One example is the cone beam computed tomography (CBCT) guided standalone linear accelerator and treatment planning system, Ethos^TM^ (Varian Medical Systems, Inc., Palo Alto, CA, USA).^9^ This novel system relies on artificial intelligence, automation and graphic processing units to offer a safe and streamlined online ART workflow.^9^ The linac is able to treat using two modes: image‐guided RT (IGRT) or ART mode. Typically IGRT mode is utilized for pre‐planned adapt on demand treatments (e.g., adapt once every five fractions). While described in more detail elsewhere,^10^, ^11^, ^12^ the general Ethos ART treatment workflow is as follows: (1) a daily CBCT is acquired, (2) AI‐ or deformable influencer contours are automatically created and reviewed by the physician (3) based on the influencer map, targets and OAR contours are generated (4) the reference plan from CT sim is recalculated on today's anatomy and a new adaptive plan re‐optimized using the reference plan objectives and (5) one of available plans is selected by physician for treatment and sent to independent QA check.

The Ethos system is equipped with a standalone treatment planning system that differs from other popular commercially available conventional planning systems. It introduces a new intelligent optimizer engine (IOE) and novel workflow for generation of the reference plan. The IOE is designed to streamline the treatment planning process with respect to conventional planning by automating the insertion of optimization parameters based on the physician's planning directives and removing planners’ direct control of the optimization space. Rather, the IOE provides the input for the optimization problem using the physician planning directives (so called "clinical goals" in the Ethos TPS) and priority rank (1‐ most important, 4‐ least important). The IOE provides an estimated dose distribution in the new Dose Preview tab.^12^ Here the planner modifies their inputted goals and priority ranks to tune their "recipe" for final plan generation. The IOE supervises this process. This streamlined workflow is helpful for many treatment sites, however it presents unique difficulties in the context of H&N treatment planning. Comparing to conventional optimization, planners can start with population based planning objectives and interactively view the resulting dose. They then adjust planning objectives during the optimization to gradually approach the optimal balance of target coverages and OAR sparing. H&N planning on Ethos is non‐intuitive to navigate the optimal dosimetric planning endpoints. This effect is compounded because the planner does not have direct control of the optimizer, but rather can only set up "clinical goals" to indirectly guide the IOE. The planner is presented with the automatically optimized plan with blinded parameters and weights. It is challenging to interpret the resulting dose distribution and "guess" how to adjust the clinical goals to further enhance plan quality. There is usually many criteria to fulfill for H&N planning and the planner may miss the optimal dose when loose planning directives are used. Furthermore, the system does not allow modification in clinical goals during online ART. Our institution has developed a practical and shareable planning strategy for online adaptive H&N treatments to mitigate the difficulties associated with choosing robust clinical goals. These clinical goals must be robust to handle potential anatomy change during ART. However, the achievable and optimal clinical goals for individual patients are usually unknown a priori.

The previously described optimization problem is not unique to online H&N ART on Ethos, but rather highlights a significant limitation to all inverse planning approaches. Manually planned H&N cases will suffer from sub‐optimal plan quality and inter‐planner variability.^13^ Tremendous efforts by the global community have been made to provide semi‐automated inverse planning solutions for all treatment specific sites using artificial intelligence. Two of the primary methods utilized are knowledge‐based planning (KBP)^14^, ^15^, ^16^, ^17^, ^18^ or in‐house deep learning techniques^19^, ^20^, ^21^ designed to guide the planner and optimization process. The overall goal of these routines is to decrease inter‐planner variability and ensure delivery of the most optimal and safe treatment plan. While these prominent solutions have demonstrated success, they are not without limitations. KBP is readily available as commercial engines exist (e.g., Varian's RapidPlan^TM^ or Pinnacle's Auto‐Plan). However, this technique is limited in its ability to determine spatial dose information.^19^ For H&N treatment planning, it has been previously demonstrated that an in‐house developed deep‐learning model that was trained with physician input provides high‐quality and consistent plans.^21^ The limitation of this model, among others, is they are not readily shareable and cannot provide a paradigm shift in H&N RT.

The purpose of this study is to help synergize the known benefits of data driven tools such as artificial intelligence and KBP in radiation treatment planning with the limitations of the Ethos IOE in the context of challenging definitive and post‐operative H&N treatments. This study will primarily focus on reference plan generation for online H&N ART. Specifically, we aim to investigate the most effective way to guide the IOE in initial reference plan generation using a (1) in‐house trained deep learning model and (2) commercially available KBP engine paired with RTOG‐based universal criteria and (3) RTOG‐based universal criteria only. This will indirectly evaluate the robustness of the IOE in initial plan generation and evaluate its sensitivity to user goals.

## 2 MATERIALS AND METHODS

### 2.1 Patient selection

Institutional Review Board (IRB) approval was obtained to use the selected twenty clinical cases for this study. The patient cohort included ten definitive and ten post‐operative cases to help fully evaluate each approach's robustness (See Table 1). The typical definitive cases included in this study were three‐level simultaneous integrated boost (SIB) dosing schemata where each target received 70 Gy, 66.5 Gy, and 56 Gy in 33 fractions. Whereas the post‐operative patients typically received a two‐dose level SIB prescription (e.g., 60 Gy and 54 Gy in 30 fractions). All patients included were clinically treated at our institution with IMRT or VMAT using a C‐arm (Truebeam/Vitalbeam, Varian Medical Systems, Inc.) or ring‐mounted gantry (Halcyon, Varian Medical Systems, Inc.) linear accelerator (linac) clinically planned in the Eclipse treatment planning system (version 16.1; Varian Medical Systems, Inc.). Each selected case was re‐planned in the Ethos TPS using a standardized 18‐field IMRT beam template. The IOE was guided by the planner using three different approaches: (1) 3D‐UNET AI dose predictor^6^ (2) RTOG universal constraint template in conjunction with commercialized KBP algorithm and (3) RTOG‐only universal constraint template (see Table 2). All approaches provided the planner with upfront clinical objectives that were input into their respective plan and are detailed in Section 2.2.

**TABLE 1:** Patient demographics

Pt No.	Clinical intent	Highest dose level (cGy)	Primary tumor location	Clinical plan technique
1	Definitive‐SIB (three dose levels)	7000	Oropharynx	17‐Field IMRT
2		7000	Hypopharynx	18‐Field IMRT
3		7000	Larynx	17‐Field IMRT
4		7000	Oropharynx	18‐Field IMRT
5		5000	Nasopharynx	17‐Field IMRT
6		7000	Oropharynx	18‐Field IMRT
7		7000	Larynx	5‐Arc VMAT
8		7000	Larynx	5‐Arc VMAT
9		7000	Oral Cavity	18‐Field IMRT
10		7000	Oropharynx	18‐Field IMRT
11	Post‐operative‐ SIB (two dose levels)	6000	Oropharynx	18‐Field IMRT
12		6000	Oral Cavity	12‐Field IMRT
13		6000	Larynx	18‐Field IMRT
14		6000	Oral Cavity	11‐Field IMRT
15		6000	Oral Cavity	14‐Field IMRT
16		6000	Oropharynx	17‐Field IMRT
17		6000	Larynx	4‐Arc VMAT
18		6000	Larynx	4‐Arc VMAT
19		6000	Larynx	5‐Arc VMAT
20		6000	Larynx	4‐Arc VMAT

*Note*: All 20 patients included in this study are shown. The cohort was equally split between definitive and post‐operative cases. Note the variable dose levels and clinical planning techniques to provide comprehensive evaluation of each method.

**TABLE 2:** Universal RTOG constraint template

Structure	Constraint
PTV + 2 cm	D_0.03cc_ < 107% of Rx
Parotid(s)	D_mean_ < 26 Gy
Brachial Plexus(s)	D_0.03cc_ < 66 Gy
Brainstem	D_0.03cc_ < 50 Gy
Cochlea(s)	D_mean_ < 35 Gy
Esophagus	D_mean_ < 30 Gy
Lips	D_mean_ < 20 Gy
Mandible	D_0.03cc_ < 73.5 Gy
Masseter muscle(s)	D_mean_ < 30 Gy
Constrictor muscle(s)	D_mean_ < 45 Gy
Pharynx	D_mean_ < 45 Gy
Submandibular gland(s)	D_mean_ < 39 Gy
Cerebral hemisphere(s)	D_mean_ < 25 Gy
Larynx	D_mean_ < 35 Gy
Optic chiasm/nerve	D_0.03cc_ < 54 Gy
PACS	D_mean_ < 45 Gy
Spinal canal	D_0.03cc_ < 45 Gy
Thyroid gland	D_mean_ < 50 Gy
Retina(s)	D_0.03cc_ < 50 Gy

*Note*: All KBP‐RTOG and RTOG plans were generated using the following constraints as a starting point. During optimization, priorities were iteratively adjusted to satisfy the dosimetric criteria. The target objectives are not shown.

### 2.2 Generalized head and neck planning approach

**FIGURE 1:**
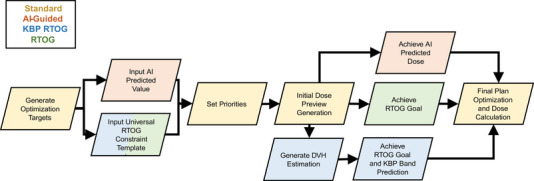
Generalized and each respective ethos H&N treatment planning workflow.

**FIGURE 2:**
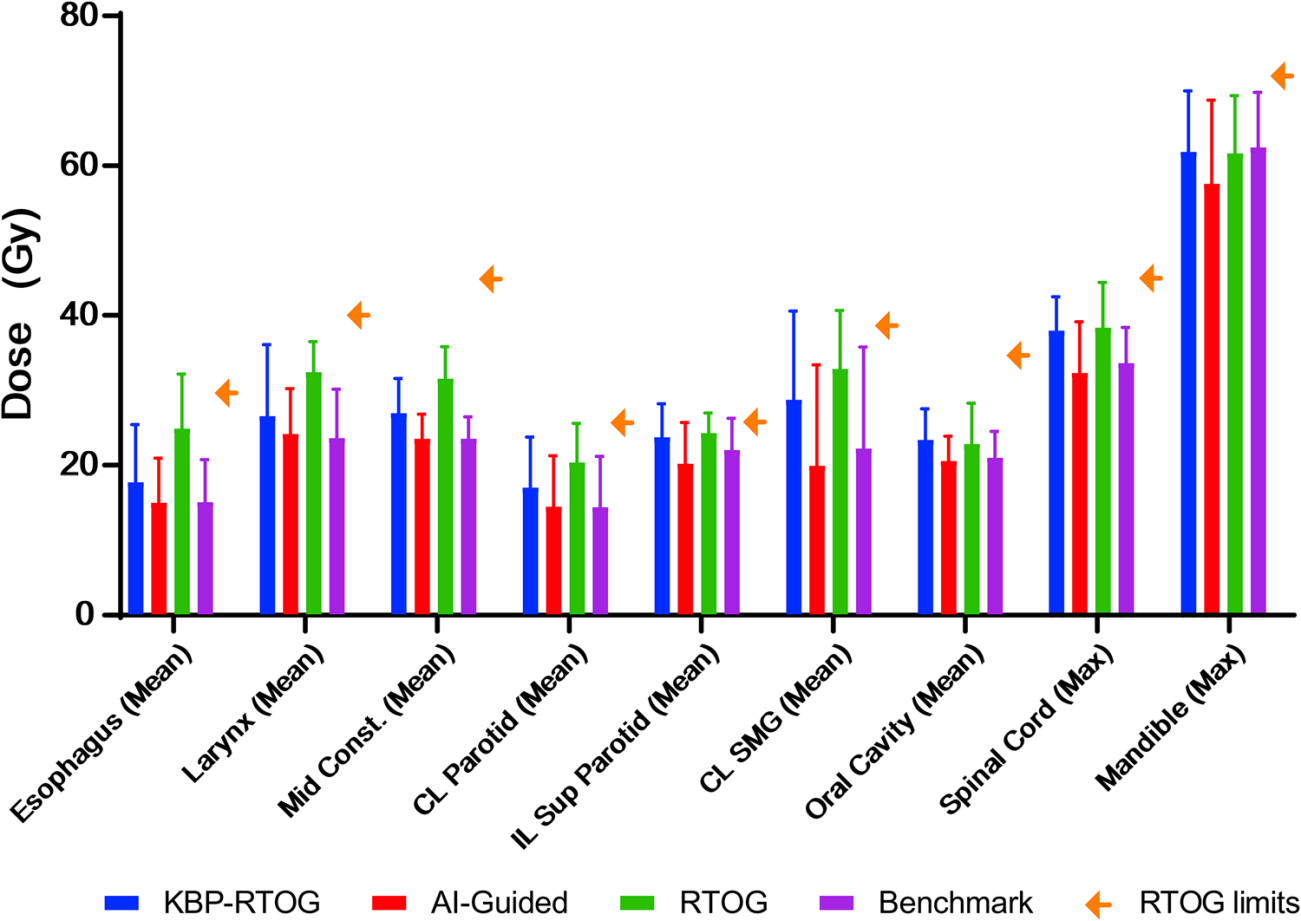
Mean and maximum OAR doses for all comparison plans including RTOG dose constraints.

Head and neck target coverage is particularly challenging in controlling the target hotspot and dose fall‐off to lower dose level targets. In addition to controlling the target dose, the presence of many DLO causes further strain on the IOE in H&N adaptive cases. To combat these issues, a novel and practical approach was developed at our institution to help planners navigate the difficulties presented by the IOE in the Ethos TPS. For general H&N adaptive planning, our institutional workflow mirrors the AI‐Guided workflow that is shown in Figure 1. First the optimization targets are generated (Section 2.2.1) and the planner then inputs the AI predicted value as the clinical goal starting point for the plan. In general, the starting priority order is as follows for Ethos treatment planning: (1) Plan hotspot (2) hard OAR constraints set by physician (3) V_100%_ coverages (4) dose fall‐off and leakage to lower dose targets (5) remaining OAR goals. This priority level is fined tuned in the dose preview tab by the planner iteratively. For this study, we designed a comparison to isolate the effects of different planning strategies on the IOE (Figure 2). Mainly, the clinical goals and optimization endpoints were variable based on planning strategy and all target generation was identical. Rather than using the 3D dose prediction as clinical goals, the three comparison plans utilized a different strategy better described in Section 2.2.2. Briefly, the KBP‐RTOG plans used the universal constraint template derived from RTOG 1016 (see Table 2) as clinical goals enabled with the KBP algorithm. Whereas the RTOG plans exclusively used the template as clinical goals.

#### 2.2.1 Target optimization

Our institution experienced difficulty in generating plans with equivalent hotspots (e.g., <107% of highest dose level) using the Ethos TPS with respect to clinically treated plans. An effective solution to mitigate this issue was to create an optimization structure that included a union of PTVs plus 2 cm margin and placing it as the highest priority objective for optimization. This effectively controls the hotspot and lessens high‐dose leakage outside of the targets. To control dose leakage between target levels, a simple and effective optimization strategy was implemented. Each lower dose level target delineated by the physician will have a corresponding optimization target. They are generated similarly but depend on the difference from the highest dose level. We found that the Ethos TPS is typically capable of producing a 3 Gy fall‐off per mm away from the target. This is best described using an example of a PTV_54Gy_ optimization (optPTV_54Gy_) structure generation with respect to the highest level PTV_60 Gy_. With a 6 Gy difference between dose levels, optPTV_54Gy_ would be generated with the following formula: PTV_54Gy_ – (PTV_60Gy_ + 0.2 mm) this is repeated for all lower corresponding dose levels.

#### 2.2.2 Organs‐at‐risk and priority

Our current clinical practice utilizes a personalized 3D dose predictor to help guide the physician in selecting dosimetric criteria. The AI allows the physician to visualize the dose distribution and guide in selection more realistic, customized objectives for each patient including their associated priorities.^6^ Planners will translate this personalized planning directive into optimization parameters and weights for plan generation. The Ethos system design was intended to not modify the planning directive, therefore no further modification is made to the planning directive, but rather the priority of the goal is adjusted. In this study, we re‐optimized the conventionally treated plans using this method in the Ethos TPS (AI‐Guided).

Another comparison plan to the clinically treated plan was generated using a commercialized KBP algorithm (RapidPlan^TM^; Varian Medical Systems, Palo Alto, CA) in conjunction with a universal RTOG‐based template in the Ethos TPS. The KBP algorithm produces dose‐volume histogram estimation bands based on previously treated cases. These KBP‐RTOG cases were optimized to satisfy two criteria: the universal RTOG constraints were met, and the dose‐volume histogram fell within its estimated bands. The planner did not receive physician input regarding IOE priority and was free to adjust as needed. Lastly, a plan was generated only using the RTOG‐based templates in order to further evaluate the effects of the KBP algorithm on the IOE and to provide guidance to clinics that are not artificial intelligence‐enabled. For the original clinical cases, the planner utilized conventional inverse planning techniques that are already well understood by the RT community.

### 2.3 Knowledge based planning and 3D dose predictor model generation

Both the KBP and 3D dose predictor model was derived from similar training data, but utilizes the data differently. Each model was trained using approximately 178 clinically treated plans that were identified to be high‐quality by a physicist. Briefly, the KBP relies on principal component analysis (PCA)‐regression to sample DVHs and geometry‐expected dose histograms (GED) of each OAR and produce an organ‐specific DVH‐estimation model. The DVH is parsed into four total regions where only the in‐field region uses PCA‐regression (if/applicable), and the remaining DVH prediction is built using the mean and standard deviation of training plans. This does not include spatial dose information and may limit the models effectiveness as many OAR share partial overlap with H&N targets.

Alternatively, the 3D dose predictor utilizes physician input to help generate the final clinical goals used for input. HD UNet is employed to predict a 3D volumetric dose distribution directly from patient target and OAR contours as input.^22^ The training of HD UNet was performed using data collected from a cohort of the above‐mentioned patients. More specifically, HD UNet was trained to minimize the mean squared error between its output and corresponding dose distributions from a clinically approved plan.^22^ A voxel‐wise uncertainty map was also generated based on the target/OAR contours representing the confidence in predicted dose.

### 2.4 Dosimetric comparison and evaluation

Each plan (e.g., AI‐guided, KBP‐RTOG, RTOG) was compared against its clinical counterpart and evaluated for target coverage, dose to OAR and plan variability. Specifically, maximum dose to highest PTV level and target coverage (all dose levels) was assessed for all plans. Hot spot assessment was performed using heterogeneity index (HI) defined as maximum dose to PTV divided by prescription dose. OAR dose criteria was assessed based on RTOG and physician guidance. Mean dose to esophagus, larynx, parotid glands, submandibular gland and oral cavity was assessed. Additionally, maximal dose to cord and mandible were recorded.

### 2.5 Modulation and deliverability evaluation

Total monitor units and subsequent modulation factor (MU/fractional dose) was calculated for each plan to assess plan complexity. Beam on time was estimated for each plan by assuming the maximum dose rate was achieved throughout the entire delivery. Additionally, independent dose verification for each plan was performed using commercialized Mobius software (Varian Medical Systems, Palo Alto, CA). All plan metrics were compared using a paired student *t*‐test with *p* < 0.05 indicating statistical significance.

## 3 RESULTS

### 3.1 Target coverage

All plans were normalized such that 95% of the highest dose target (e.g., 70 Gy for a standard SIB three‐dose level schemata) of receives 100% of the prescription dose. To evaluate near target minimum coverage, D_99%_(%) was recorded for each PTV dose level. Typically, definitive cases utilized a three‐dose level schemata whereas post‐operative cases used two dose levels. For clarity, these dose levels were recorded as PTV_High_, PTV_Mid_, and PTV_Low_ dose levels. All *p*‐values presented in this section are with respect to clinical benchmark plans. PTV_High_ D_99%_ on average was 95.3% ± 6.6% (*p* = n.s.), 94.53% ± 2.9% (*p* < 0.001), 97.0% ± 1.5% (*p* = n.s.) and 97.1% ± 2.0% for KBP‐RTOG, AI‐guided, RTOG and benchmark plans, respectively. Note that AI‐guided plans target coverage were statistically significantly different than benchmark, which may explain OAR sparing differences as the IOE is more aggressively driving optimization. For PTV_Low_ D_99%_, recorded averages were 97.9% ± 4.1% (*p* = n.s.), 95.3% ± 3.8% (*p* = n.s.), 98.1% ± 3.1% (*p* = n.s.), and 96.9% ± 2.5% for KBP‐RTOG, AI‐guided, RTOG and benchmark plans, respectively. PTV_Mid_ is not reported in this section as many post‐operative cases did not have a third dose level, ultimately lowering its sample power. The average HI was 1.06 ± 0.03 for each set of plans and exhibited no statistical significance with respect to benchmark clinical plans.

### 3.2 OAR sparing

As a general trend, AI‐guided plans demonstrated the highest plan quality versus KBP‐RTOG and RTOG as they typically increased OAR sparing with no to minimal cost of target coverage, hot‐spot or modulation (see Figure 2). The mean ipsilateral superficial parotid (*p* = 0.004) and mandible maximum dose (*p* = 0.04) were improved when compared to benchmark plans on average 1.8 Gy and 0.61 Gy respectively for AI‐guided plans. All other OAR differences were statistically insignificant. KBP‐RTOG plans showed statistically significant differences (typically increased) with benchmark plans for the mean larynx (*p* < 0.001), mean contralateral parotid (*p* < 0.002), mean contralateral submandibular gland (*p* = 0.005) and spinal cord maximal dose (*p* = 0.002). The RTOG plans exhibited statistically significant worsened OAR sparing differences for mean esophagus (*p* < 0.001), mean larynx (*p* < 0.001), mean constrictor (*p* = 0.03), mean contralateral parotid (*p* < 0.001), mean ipsilateral superior parotid (*p* = 0.006), mean contralateral submandibular gland (*p* = 0.006) and maximum spinal cord dose (*p* = 0.003). While many of the OARs displayed significantly statistical differences for KBP‐RTOG and RTOG versus benchmark, this result is expected as the benchmark plans were generated using a similar conventional AI‐guided workflow. Moreover, on average all plans satisfied RTOG criteria indicating each approach is clinically feasible and will deliver a safe treatment.

### 3.3 Plan deliverability

Plan deliverability metrics are shown in Table 3. As a general trend RTOG plans were the least modulated whereas the AI‐guided plans showed the highest modulation. This can be explained as the AI‐guided plans utilized the most demanding constraints during planning and showed better OAR sparing compared to other approaches. While this complexity is statistically significant, it may not be clinically significant as the Ethos system is capable of 800 MU/min dose rate ultimately only adding on 1.1 min of average beam‐on time. All plans additionally passed gamma‐analysis using a 3% dose difference and 2 mm distance‐to‐agreement criteria indicating plans can be accurately delivered.

**TABLE 3:** Plan deliverability metrics

	Total MU (MU)	Mod. factor (MF)	Esti beam‐on‐time (min)	Mobius γ‐passing criteria (3%/2 mm)
AI‐guided	2632 ± 289**	13.1 ± 1.4**	3.6 ± 0.5**	94.5 ± 1.5*
KBP‐RTOG	2447 ± 389	12.2 ± 1.9	3.4 ± 0.7	94.9 ± 1.6
RTOG	2303 ± 260	11.5 ± 1.3	3.1 ± 0.5	94.6 ± 1.8*
Benchmark	2442 ± 384	12.2 ± 4.3	2.5 ± 0.95	96.1 ± 1.7

*Note*: Select plan deliverability metrics for all comparison cases including statistical significance with respect to benchmark cases. VMAT plans were excluded from Benchmark data.

^*^ *p* < 0.05, ^**^ *p* < 0.001.

### 3.4 Example patient

**FIGURE 3:**
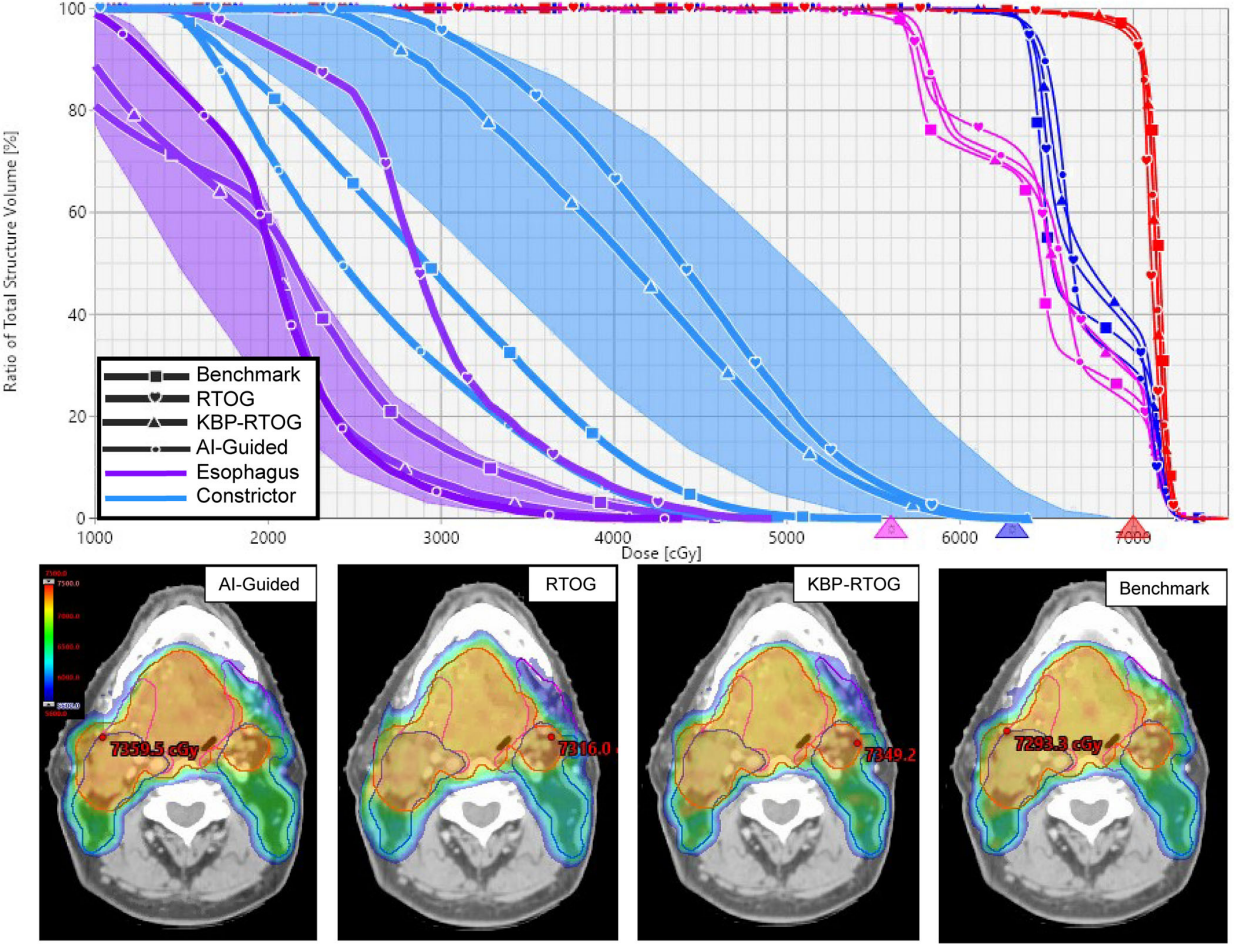
Single axial plane and corresponding dose‐volume‐histogram for example patient.

An axial single plane of an example patient that represents the average finding of each plan cohort with corresponding DVH is shown in Figure 3. The isodose color‐wash (blue = 56 Gy, red = 70 Gy) overall show comparable trends across all plans. AI‐guided appears to be more conformal with respect to other comparison plans. The DVH displays the constrictor and DVH‐estimate (light blue), Esophagus (purple) PTV_70Gy_ (red), PTV_63Gy_ (dark blue) and PTV_56Gy_ (pink). All targets receive similar coverage despite noticeable differences in constrictor and esophagus sparing.

This example DVH highlights two important observations regarding the strength of the KBP model within the IOE. The KBP model can over predict the dose as seen in the constrictor estimation despite using similar training data as the AI‐guided model. As a consequence of this predication, the KBP‐RTOG falls within the estimation whereas the AI‐guided Ethos and benchmark clinical plans achieve better sparing with no loss to coverage. Alternatively, for the esophagus, all plans except for the RTOG‐only plan fall within the DVH‐estimation band. This indicates one of the strengths of using either a KBP or deep‐learning model in the context of H&N ART.

## 4 DISCUSSION

This study evaluated the Ethos IOE for adaptive H&N planning using three different planning approaches with respect to clinically treated plans from either a C‐arm or ring‐mounted linac planned in the commercially available Eclipse treatment planning system. In general, it was found that utilizing an advanced AI‐guided approach produces superior plan quality in the Ethos IOE. However, for clinics that are not able to generate a deep‐learning model, a commercial KBP algorithm or using population‐based constraint templates will yield acceptable and safe plan quality. Our institution's benchmark data was driven using AI, therefore it was necessary to utilize AI in the IOE to reproduce plan quality. A few important observations regarding the IOE warrants additional discussion. Compared to Eclipse, the IOE is more sensitive to clinical goal input, that is, the template constraint and final dose are typically comparable. This implies that for this system it is not appropriate to request unrealistic planning goals as it will attempt to satisfy that criteria and significantly lower target coverage. Another important distinction between the Ethos IOE and Eclipse TPS is the handling of the DVH‐estimation weighting. In Eclipse, the user has control of the line dose constraint and more effectively drives the entire plan optimization. The line‐dose constraint is The KBP model utilizes a line‐dose constraint to help drive the DVH to within its estimation band. This is accomplished by inserting upper dose objectives (i.e., a line) at the lower standard deviation of the DVH‐estimation band produced by the KBP. However, in Ethos, the original objective from the universal template is weighted significantly heavier than the line dose objective. Lastly, the maximum dose is not directly controlled using the line‐objectives generated by the KBP algorithm. We recommend adding more demanding maximum dose clinical objectives to satisfy institutional criteria, if needed. Therefore, this partially explains why the AI‐guided plans outperform the KBP‐enabled comparison plans.

At time of preparation of this manuscript, there were no other available studies that discuss optimization strategies in H&N adaptive radiotherapy. This is likely due to the challenging requirements to plan a high‐quality H&N reference plan in conjunction with limited system users. Regardless of final clinical goal input, this work highlights a fundamental planning approach that can be used as a starting point for all H&N adaptive planning (see Table 4).

**TABLE 4:** Generalized planning strategy for ethos H&N adaptive radiotherapy planning

Structure	Goal	Order	Formula/Note
PTV_D_max_	D_0.03cc_ < 107% Rx	Highest P1	(All PTV + 2 cm) minus body
Hard constraint OAR	MD defined clinical goal	P1	–
PTV	V_100%_ ≥ 95%	P1	–
Small volume Tier 1 OAR	MD defined clinical goal	P1	–
PTV	V_95%_ ≥ 99%	P1	–
Remaining tier 1 OAR	MD defined clinical goal	P1	–
optPTV/dose‐tuning	D_10%_ <100% RXlow D_0.03cm3_ <105% RXlow	P1	[PTV_low_ ‐ (PTV_high_+ x mm)] x = (Rx_high_‐Rx_low_)/3 + 1 mm
Tier 2 OAR	MD defined clinical goal	P2‐P3	Only P1 and P2 goals will allow be taken into account during online ART

*Note*: Generalized planning strategy regardless of clinical goal criteria is displayed. This serves as a starting point for H&N initial reference plan generation and can be adjusted on a patient‐specific basis.

The IOE struggles to control the PTV maximum hotspot in plans, indicating the necessity to place the hot spot tuning structure to the number one priority followed by hard constraint OAR. PTV D_95%_ coverage is the next priority followed by tier 1 OARs with a small volume. It was noticed Ethos poorly crops dose in small volume OAR unless it is heavily prioritized by the planner during optimization. In select cases, these special OARs can be placed higher in priority without impact to PTV coverage. Lastly, PTV dose tuning structures are placed in the first priority level for the plan. All remaining MD defined OAR (tier 2) clinical goals are adjusted in lower priority levels (e.g., P2, P3). This same fundamental approach may be applicable to other sites on Ethos and different ART systems (e.g., MR‐guided RT).

Another important observation that warrants discussion is the higher modulation factor for both Ethos and benchmark plans. The Halcyon/Ethos system is equipped with a new stacked and staggered MLC design when compared to conventional C‐arm linacs.^23^ This new design offers lower leaf leakage and transmission that allows the system to “paint” the target dose by increasing the modulation for minimal costs. Our institution uses 18‐field IMRT which further contributes to higher MUs rather than a more traditional 12‐field approach. The latter approach is not utilized in our clinic as it does not provide consistent plan quality and is further worsened as the geometry complexity of cases increases. Fortunately, our gamma passing rate is still acceptable and provides confidence in our treatment delivery accuracy.

One important limitation to this study is that our benchmark clinical plans were generated with an AI‐guided workflow using our in‐house 3D dose predictor. Therefore, our benchmark data was generated with more stringent criteria than population based metrics in most cases. However, the RTOG criteria is included in the dosimetric evaluation section to allow for comparisons to the national standard for H&N treatments. This study does not also evaluate the robustness of these approaches during online adaptation and may warrant further evaluation in future. In parallel to this work, ongoing studies that utilize the 3D dose predictor for initial plan evaluation are simulating daily adaptive radiotherapy with significantly reduced margins in definitive H&N cancer.^24^ Moreover, another ongoing study is retroactively analyzing online adaption in an adjuvant setting.^25^ Both studies preliminarily indicate the 3D dose predictor will generate robust clinical goals that will generate acceptable online plans.

## 5 CONCLUSION

This study reviews and demonstrates three practical approaches to generating initial reference plans for both definitive and post‐operative head and neck adaptive treatments using the Ethos system. Two of these strategies utilized common AI‐guidance, deep‐learning and knowledge‐based planning, whereas the final strategy solely used a universal constraint template based on national standard population‐based RTOG metrics. All approaches generate a safe‐plan that satisfied RTOG accepted criteria, however the AI‐deep learning guided model generated the most optimal initial reference plan. Additionally, the new IOE was evaluated and shown to be more sensitive than the conventional Varian Eclipse treatment planning system. The purpose of this work was not to recommend one method versus the other, but rather to provide guidance to the RT community as more clinics become adaptive therapy enabled. Further study to evaluate each workflow's online robustness and sensitivity is warranted.

## AUTHOR CONTRIBUTION

Justin Visak and Mu‐Han Lin designed the study. Justin Visak, Mu‐Han Lin, Tingliang Zhang re‐planned clinical cases. Dan Nyugen, Mu‐Han Lin and Steve Jiang designed machine‐learning models. Enobong Inam, Boyu Meng and Siqiu Wang performed data collection and analysis. Justin Visak and Mu‐Han Lin drafted first iteration of manuscript. Vladimir Avkshtol, David Sher and Dominic Moon provided clinical supervision and input to the project. All authors revised and approved the final manuscript.

## CONFLICT OF INTEREST STATEMENT

No conflicts of interest.

## Data Availability

The data that support the findings of this study are available from the corresponding author upon reasonable request.
